# Supplementary material for: Derivation and validation of an epigenetic frailty risk score in population-based cohorts of older adults
Source: Nat Commun. 2022 Sep 7;13:5269. doi: 10.1038/s41467-022-32893-x (PMC9450828; doi:10.1038/s41467-022-32893-x)
Supplement: Supplementary file 1 — Supplementary Information [file 41467_2022_32893_MOESM1_ESM.pdf]

Supplementary Table 1. Associations of previously reported frailty-related CpGs with frailty index in subset III

CpG site	Linear mixed regression	
	Coefficient (95% CI) [a]	*P*-value [b]
cg14753356	-2.73 (-4.29--1.16)	0.001
cg19589396	-1.84 (-2.86--0.82)	0.000
cg05673882	-1.65 (-2.67--0.62)	0.002
cg23667432	-1.50 (-2.66--0.35)	0.011
cg07826859	-1.43 (-2.83--0.04)	0.044
cg01127300	-1.27 (-2.16--0.38)	0.005
cg02657160	-1.23 (-2.15--0.32)	0.009
cg19859270	-1.17 (-2.16--0.18)	0.021
cg25189904	-0.44 (-1.38-0.50)	0.361
cg13072214	-0.32 (-1.32-0.69)	0.538
cg15709766	0.25 (-0.58-1.08)	0.558
cg16421157	0.30 (-0.56-1.17)	0.492
cg00861009	0.31 (-0.61-1.22)	0.512
cg18314882	0.43 (-0.46-1.31)	0.346
cg17588578	0.57 (-0.29-1.44)	0.193

Abbreviations: CI, confidence interval.

[a] Coefficients of linear mixed regression and *P* values of per standard deviation in methylation with per % of frailty index. The models were adjusted for age, sex, leukocyte composition, batch, smoking status (never smoker, former smoker, current smoker), and alcohol consumption (grams per day).

[b] All *P* values are two-sided with adjustments.

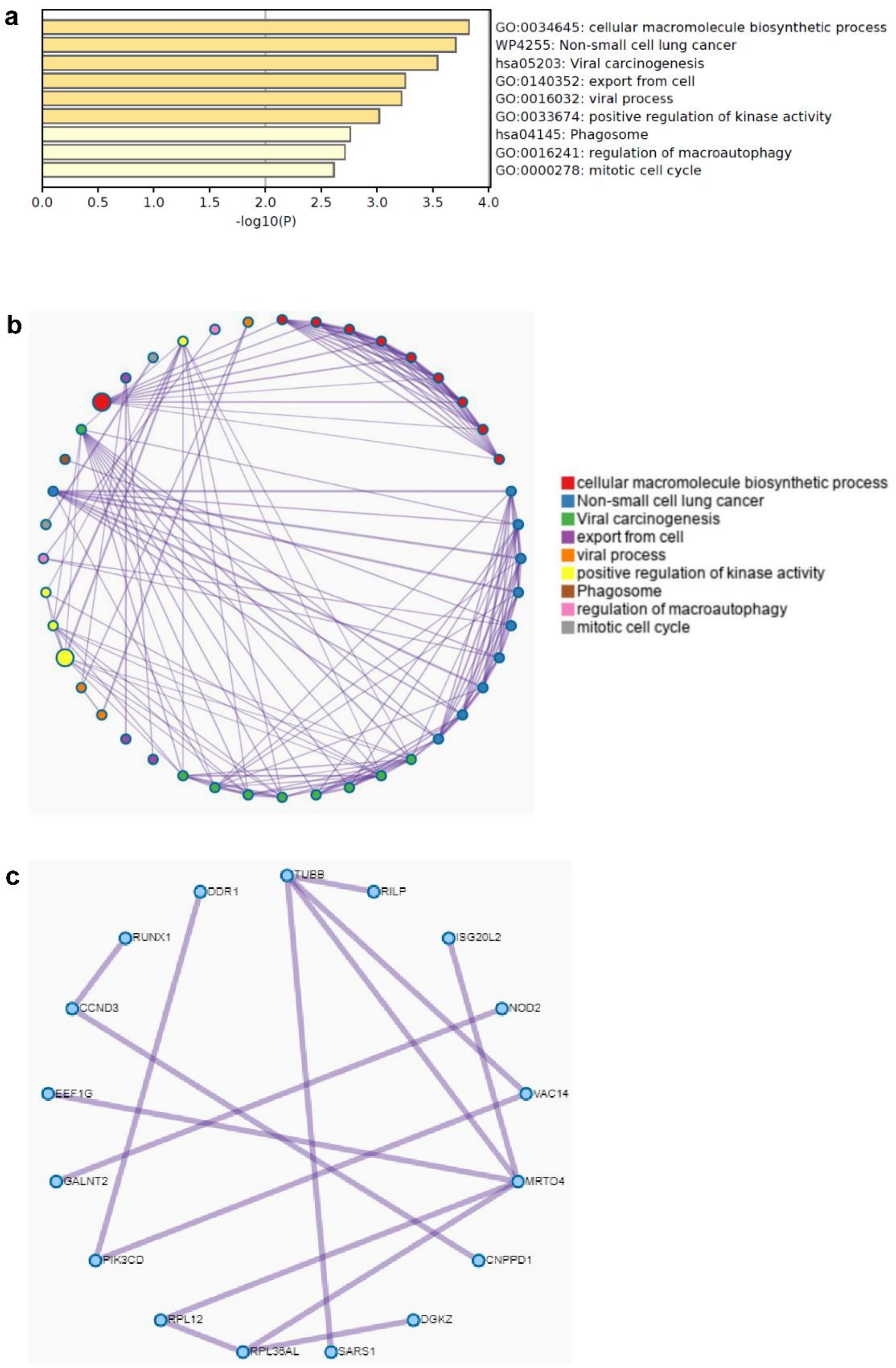

Supplementary Figure 1. Pathway enrichment and protein-protein interaction (PPI) network analysis of target genes of frailty-related CpGs.

a. Network of enriched terms colored based on *P* value (two-sided without adjustments).

b. Demonstration of enriched network layout by circle nodes for each terms.

c. PPI network and MCODE components identified in the gene lists.

Supplementary Table 2. Associations of eFRS at baseline with frailty index at baseline and each follow-up

	N	Increase in FI (CI) in % per 1 SD increase of eFRS [a]			
		**Model 1**	***P***	**Model 2**	***P*** [b]
**Subset III (ESTHER study)**					
FI-BL	538	1.65 (0.58-2.72)	<0.01	1.78 (0.66-2.90)	<0.01
FI-2Y	507	1.84 (0.65-3.02)	<0.01	2.11 (0.85-3.37)	<0.01
FI-5Y	449	2.13 (0.81-3.45)	<0.01	1.96 (0.52-3.40)	0.01
FI-8Y	369	2.16 (0.57-3.74)	0.01	1.94 (0.24-3.64)	0.03
FI-11Y	258	1.67 (-0.27-3.62)	0.09	1.55 (-0.47-3.57)	0.14
**Subset IV (KORA-Age study)**					
FI-BL	1007	1.42 (0.64-2.20)	<0.01	1.28 (0.51-2.05)	<0.01
FI-4Y	771	1.97 (0.93-3.01)	<0.01	1.72 (0.67-2.76)	<0.01
FI-8Y	585	1.55 (0.17-2.94)	0.03	1.27 (-0.12-2.66)	0.07

Abbreviations: eFRS, epigenetic frailty risk score; FI, frailty index; CI, confidence interval; SD, standard deviation; FI-BL, baseline frailty index; FI-2Y, 2-year follow-up frailty index; FI-4Y, 4-year follow-up frailty index; FI-5Y, 5-year follow-up frailty index; FI-8Y, 8-year follow-up frailty index; FI-11Y, 11-year follow-up frailty index.

Model 1, adjusted for age, sex, leukocyte composition, and batch.

Model 2, similar as model 1, additionally adjusted for baseline smoking status (never smoker, former smoker, current smoker), and alcohol consumption (grams per day).

[a] Coefficients of linear mixed regression. Estimated change (95% confidence interval) of frailty index (expressed in %) per increase of frailty risk score by one standard deviation (SD=0.03).

[b] All *P* values are two-sided with adjustments.

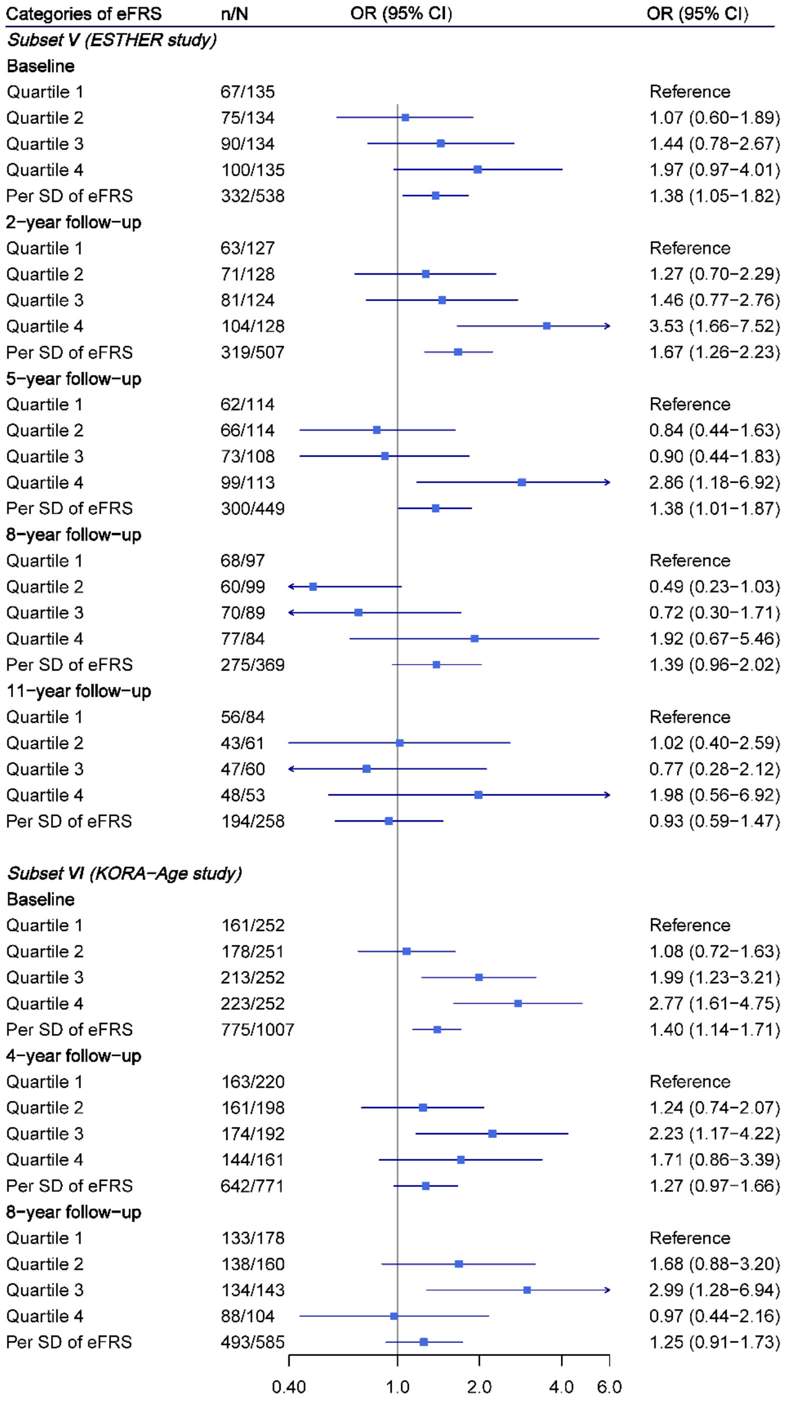

Supplementary Figure 2. Association of epigenetic frailty risk score with being pre-frail or frail at follow-ups

Abbreviations: eFRS, epigenetic frailty risk score; OR, odds ratio; CI, confidence interval; SD, standard deviation.

Vertical ticks within the blue boxes and horizontal lines show the OR and 95% CI. Models were adjusted for age, sex, leukocyte composition, batch, baseline smoking status (never smoker, former smoker, current smoker), and alcohol consumption (grams per day).

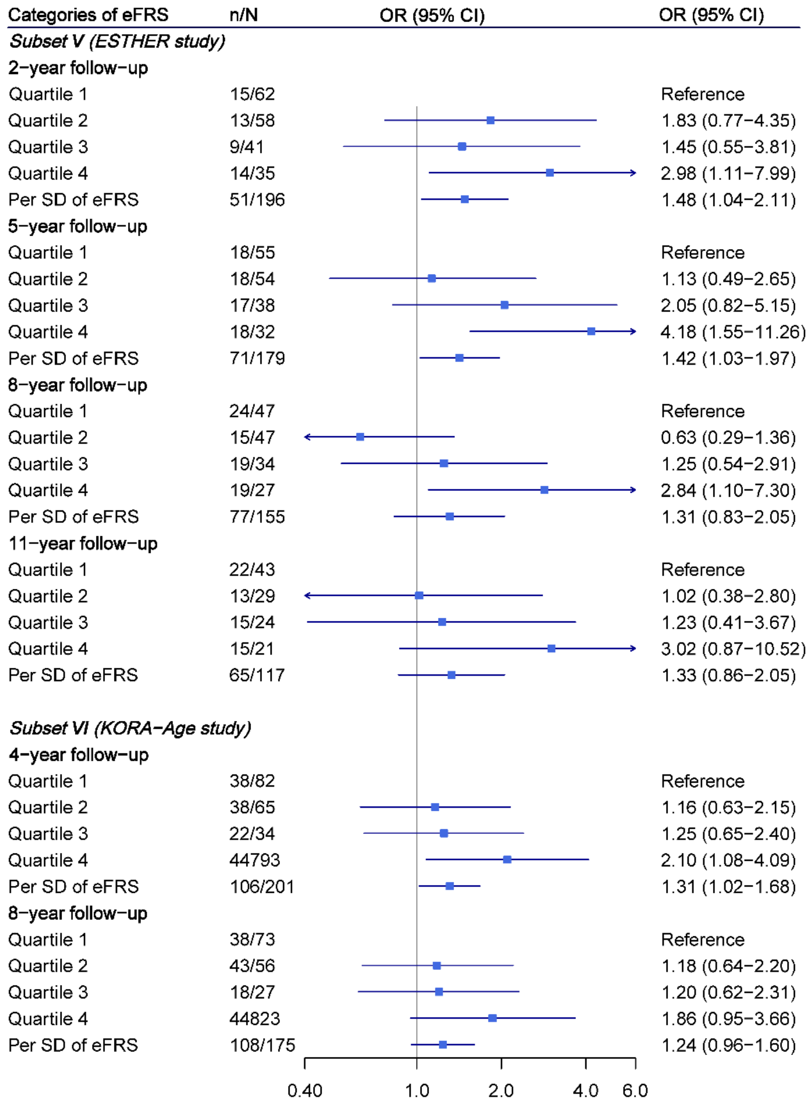

Supplementary Figure 3. Association of eFRS with being pre-frail or frail among participants who were being non-frail at baseline

Abbreviations: eFRS, epigenetic frailty risk score; OR, odds ratio; CI, confidence interval; SD, standard deviation.

Vertical ticks within the blue boxes and horizontal lines show the OR and 95% CI. Models were adjusted for age, sex, leukocyte composition, batch, baseline smoking status (never smoker, former smoker, current smoker), and alcohol consumption (grams per day).

Supplementary Table 3. Association of eFRS with frailty at follow-ups by models adjusted for different models in ESTHER

	OR (95% CI, per SD of eFRS)	
	**Models adjusted for smoking status [a]**	**Models adjusted for Mass 13-CpGs model for smoking [b]**
**Association of eFRS with being pre-frail or frail**		
Baseline	1.38 (1.05-1.82)	1.46 (1.12-1.91)
2-year follow-up	1.67 (1.26-2.23)	1.52 (1.15-2.01)
5-year follow-up	1.38 (1.01-1.87)	1.35 (1.00-1.83)
8-year follow-up	1.39 (0.96-2.02)	1.44 (0.93-2.22)
11-year follow-up	0.93 (0.59-1.47)	1.13 (0.72-1.78)
**Association of eFRS with being frail**		
Baseline	1.94 (1.31-2.89)	1.99 (1.25-3.17)
2-year follow-up	1.64 (1.15-2.35)	1.74 (1.10-2.88)
5-year follow-up	1.48 (1.07-2.04)	1.44 (0.99-2.15)
8-year follow-up	1.29 (0.91-1.84)	1.27 (0.71-2.24)
11-year follow-up	1.34 (0.87-2.08)	1.41 (0.92-2.17)

Abbreviations: eFRS, epigenetic frailty risk score; OR, odds ratio; CI, confidence interval; SD, standard deviation.

[a] Models were adjusted for age, sex, leukocyte composition, batch, baseline smoking status (never smoker, former smoker, current smoker), and alcohol consumption (grams per day).

[b] Models were adjusted for age, sex, leukocyte composition, batch, Mass 13-CpGs model for smoking, and alcohol consumption (grams per day).

Supplementary Table 4. Associations of AgeAccGrim at baseline with frailty index at baseline and each follow-up in ESTHER

	N	Increase in FI (CI) in % per 1 SD increase of AgeAccGrim [a]			
		**Model 1**	***P*** [b]	**Model 2**	***P*** [b]
FI-BL	538	1.74 (0.96-2.52)	<0.01	1.73 (1.10-2.35)	<0.01
FI-2Y	507	1.53 (0.68-2.39)	<0.01	1.78 (1.10-2.47)	<0.01
FI-5Y	449	1.28 (0.29-2.27)	0.01	1.67 (0.88-2.46)	<0.01
FI-8Y	369	1.78 (0.73-2.82)	<0.01	1.88 (1.02-2.73)	<0.01
FI-11Y	258	2.05 (0.59-3.52)	0.01	2.17 (0.95-3.39)	<0.01

Abbreviations: AgeAccGrim, GrimAge age acceleration; FI, frailty index; CI, confidence interval; SD, standard deviation; FI-BL, baseline frailty index; FI-2Y, 2-year follow-up frailty index; FI-4Y, 4-year follow-up frailty index; FI-5Y, 5-year follow-up frailty index; FI-8Y, 8-year follow-up frailty index; FI-11Y, 11-year follow-up frailty index.

Model 1, adjusted for age, sex, leukocyte composition, and batch.

Model 2, similar as model 1, additionally adjusted for baseline smoking status (never smoker, former smoker, current smoker), and alcohol consumption (grams per day).

[a] Estimated change (95% confidence interval) of frailty index (expressed in %) per increase of GrimAge age acceleration by one standard deviation (SD=4.72).

[b] All *P* values are two-sided with adjustments.

Categories of eFRS	n/N	OR (95%CI)
Subset III (ESTHER study)		
Baseline		
Quartile 1	74/134	Reference
Quartile 2	77/135	1.29 (0.75−2.19)
Quartile 3	91/135	2.35 (1.29−4.29)
Quartile 4	90/134	2.73 (1.34−5.57)
Per SD of AgeAccelGrim	332/538	1.40 (1.04−1.89)
2−year follow−up		
Quartile 1	67/125	Reference
Quartile 2	79/129	1.20 (0.69−2.08)
Quartile 3	83/128	1.99 (1.07−3.68)
Quartile 4	90/125	2.11 (1.01−4.39)
Per SD of AgeAccelGrim	319/507	1.37 (1.04−1.82)
5−year follow−up		
Quartile 1	60/113	Reference
Quartile 2	82/117	1.57 (0.88−2.82)
Quartile 3	83/111	3.17 (1.59−6.31)
Quartile 4	75/108	2.63 (1.18−5.85)
Per SD of AgeAccelGrim	300/449	1.52 (1.10−2.09)
8−year follow−up		
Quartile 1	61/97	Reference
Quartile 2	69/96	1.53 (0.78−2.99)
Quartile 3	77/93	2.05 (0.93−4.52)
Quartile 4	68/83	2.38 (0.92−6.17)
Per SD of AgeAccelGrim	275/369	1.46 (0.99−2.16)
11−year follow−up		
Quartile 1	48/72	Reference
Quartile 2	54/72	1.57 (0.65−3.82)
Quartile 3	51/65	1.92 (0.63−5.82)
Quartile 4	41/49	2.36 (0.64−8.73)
Per SD of AgeAccelGrim	194/258	1.78 (0.99−3.21)

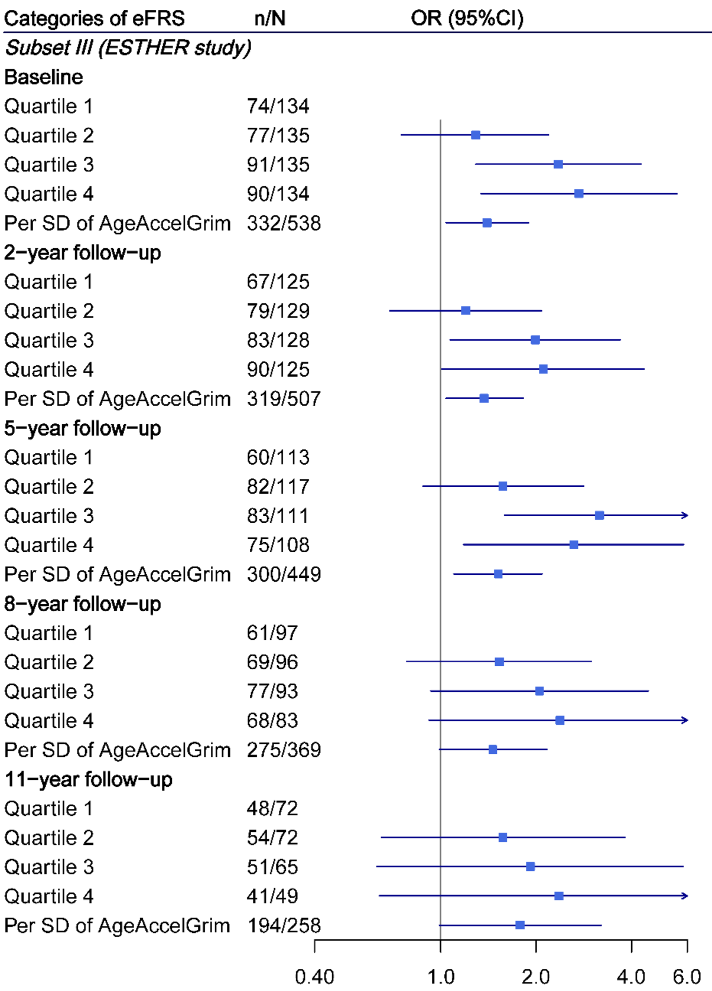

Supplementary Figure 4. Association of AgeAccGrim with being pre-frail or frail at follow-ups in ESTHER

Abbreviations: AgeAccGrim, GrimAge age acceleration; OR, odds ratio; CI, confidence interval; SD, standard deviation.

Vertical ticks within the blue boxes and horizontal lines show the OR and 95% CI. Models were adjusted for age, sex, leukocyte composition, batch, baseline smoking status (never smoker, former smoker, current smoker), and alcohol consumption (grams per day).

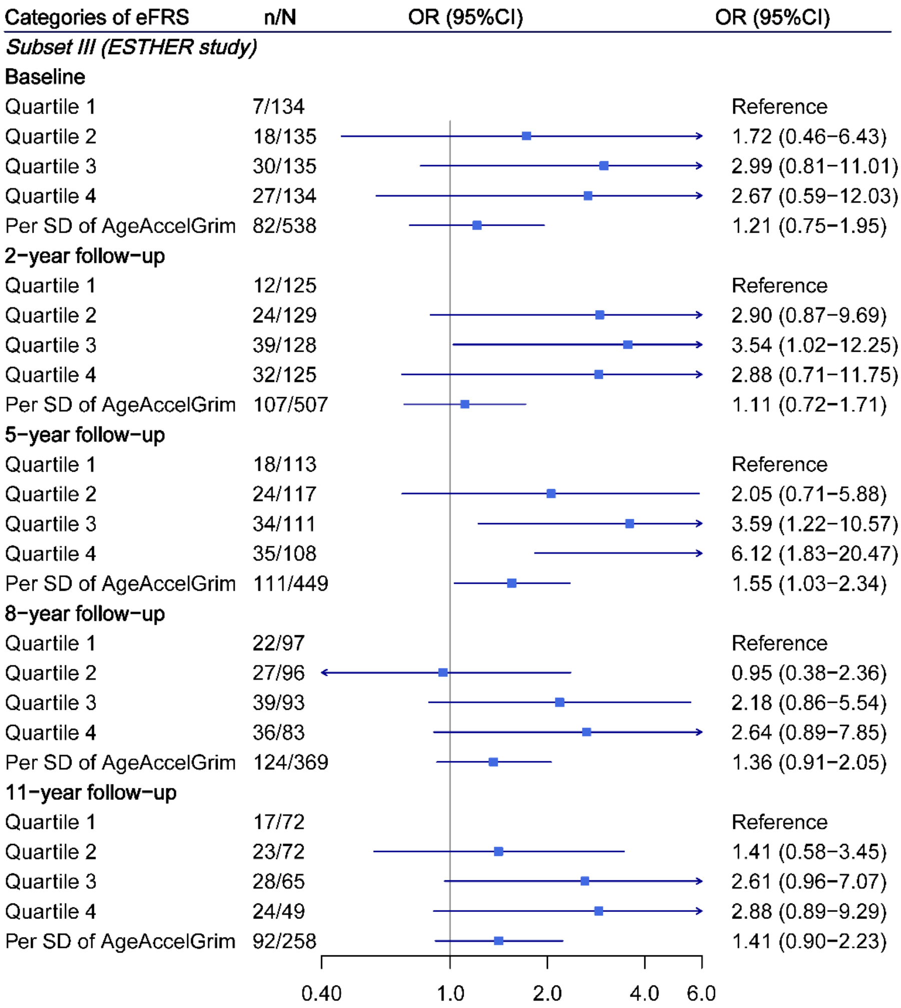

Supplementary Figure 5. Association of AgeAccGrim with being frail at follow-ups in ESTHER

Abbreviations: AgeAccGrim, GrimAge age acceleration; OR, odds ratio; CI, confidence interval; SD, standard deviation.

Vertical ticks within the blue boxes and horizontal lines show the OR and 95% CI. Models were adjusted for age, sex, leukocyte composition, batch, baseline smoking status (never smoker, former smoker, current smoker), and alcohol consumption (grams per day).

Supplementary Table 5. Deficits included in the frailty index calculation in ESTHER

Variable name	Coding
**History of diseases** (7 items)	
Coronary artery disease	1=Yes, 0=No
Heart failure	1=Yes, 0=No
Diabetes	1=Yes, 0=No
Cancer	1=Yes, 0=No
Glaucoma	1=Yes, 0=No
Cataract	1=Yes, 0=No
Parkinson	1=Yes, 0=No
**History of major disease events** (4 items)	
Myocardial infarction	1=Yes, 0=No
Stroke	1=Yes, 0=No
Joint replacement	1=Yes, 0=No
Femoral neck fracture	1=Yes, 0=No
**Drugs** (10 items)	
Anti-hypertensives	1=Yes, 0=No
Lipid lowering drugs	1=Yes, 0=No
Vasodilatators	1=Yes, 0=No
Heart glycosides	1=Yes, 0=No
Prescribed aspirin	1=Yes, 0=No
Anti-ostoporotic drugs	1=Yes, 0=No
Anxiolytics	1=Yes, 0=No
Sedatives	1=Yes, 0=No
Anti-dementive drugs	1=Yes, 0=No
Drugs against prostatic hyperplasia and incontinence	1=Yes, 0=No
**Difficulties in activities of daily living** (6 items)	
Difficulties in moderate activities	1=Yes, 0.5= Limited, 0=No
Difficulties in climbing several flights of stairs	1=Yes, 0.5= Limited, 0=No
Limits in normal work or activities due to pain	1=Extremely limited, 0.75=Quite a lot, 0.5=Moderate, 0.25=A bit, 0=Not at all
Limits in certain work or activities	1=Yes, 0=No
Limits in contact with others	1=Always, 0.75=Mostly, 0.5=Sometimes, 0.25=Rare, 0=Never
Limits in activities due to mental health	1=Yes, 0=No
**General health** (1 items)	
General self-rated health	1=Poor, 0.75=Less good, 0.5=Good, 0.25=Very good, 0=Excellent
**Life-style related factors** (3 items)	
Underweight	0=BMI≥20, 1=BMI< 20
Overweight	0=BMI <30, 0.5=BMI 30-35, 1=BMI≥ 35
Lack of vigorous physical activity	0= ≥ 0 hour/week, 1= 0 hour/week

Abbreviation: BMI, body mass index.

Supplementary Table 6. Deficits included in the frailty index calculation in KORA-Age

Disability Measures	Diseases	Signs and Symptoms
1. Arising [a]<br>2. Hygiene [a]<br>3. Eating [a]<br>4. Gripping [a]<br>5. Dressing [a]<br>6. Grooming [a]<br>7. Walking [a]<br>8. Taking stairs [a]<br>9. Reaching over one's head [a]<br>10. Stooping [a]<br>11. Shopping [a]<br>12. Getting in and out of cars [a]<br>13. Housework and gardening [a]	14. Lung Disease [b]<br>15. Joint Disease [b]<br>16. Heart complaints / diseases [b]<br>17. Stroke [b]<br>18. Cancer [b]<br>19. Diabetes Mellitus [b]<br>20. Eyes Disease [b]<br>21. Anxiety [b]<br>22. Depression [b]<br>23. Hip Fractures in the last 24 months [b]	24. Cognitive deficits [c]<br>25. Malnutrition [d]<br>26. Falls in the last 12 months [b]<br>27. Weight Loss over 5kg in the last 6 months [b]<br>28. Dizziness in the last 12 months [b]<br>29. Fatigue in the last two weeks (never felt energetic and active) [b]<br>30. Sleep deficits [e]<br>31. Hospital admission in the last 12 months [b]<br>32. Pain [f]<br>33. Hypertension [b]

[a] Items with four answer options on a Likert scale, recoded as follows: Without difficulties = 0 (no deficit), some difficulties = 0.33, considerable difficulties = 0.67, Impossible = 1 (full deficit).

[b] Binary Item, coded as 0 (deficit not indicated as present by the participant) or 1 (deficit indicated as present by the participant).

[c] Classification according to Telephone Interview for Cognitive Status-modified (TICS-m) [1] into not impaired, mildly impaired, and impaired and coded as not impaired = 0 (no deficit), mildly impaired = 0.5, impaired = 1 (full deficit).

[d] SCREEN II (short version of the "Seniors in the community: risk evaluation for eating and nutrition, verion II") Score ≥ 43 coded as 0 (no deficit), SCREEN Score ≤ 42 coded as 1 (full deficit) [2].

[e] 0 (no deficit) for nearly never having difficulties falling asleep and sleeping through the night, 0.5 deficit points for sometimes having difficulties either falling asleep or sleeping through the night, 1 deficit point (full deficit) for often having difficulties either falling asleep or sleeping through the night.

[f] Classification as measured by EQ-5D [3,4] pain item: no pain = 0 (no deficit), slight pain = 0.25, moderate pain = 0.5, severe pain = 0.75, extreme pain =1 (full deficit).

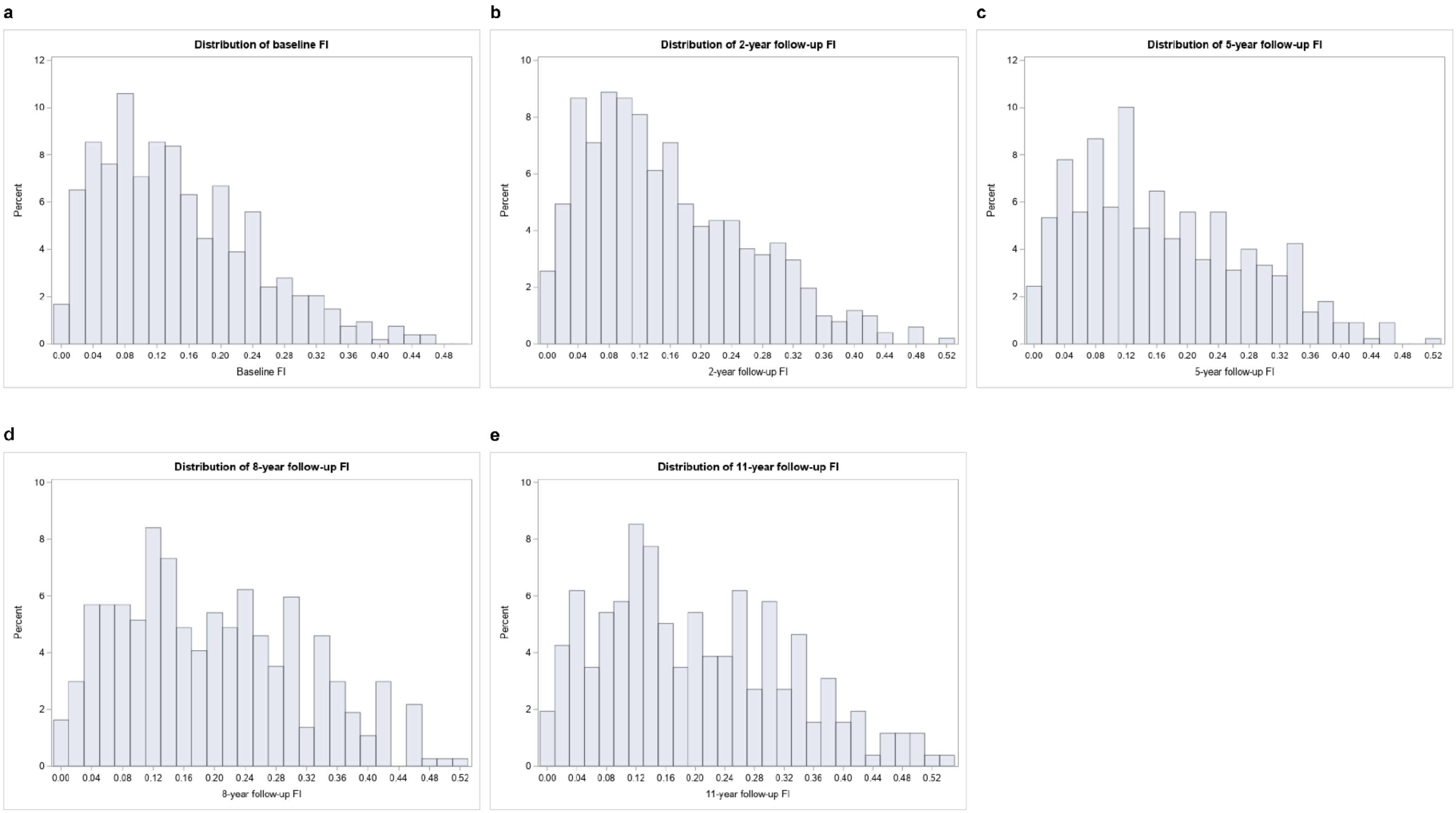

Supplementary Figure 6. Distributions of FI at baseline and each follow-up in ESTHER

Abbreviation: FI, frailty index.

a. Distribution of baseline FI.

b. Distribution of 2-year follow-up FI.

c. Distribution of 5-year follow-up FI.

d. Distribution of 8-year follow-up FI.

e. Distribution of 11-year follow-up FI.

Supplementary References

1 Knopman, D. S. *et al.* Validation of the telephone interview for cognitive status-modified in subjects with normal cognition, mild cognitive impairment, or dementia. *Neuroepidemiology* **34**, 34-42 (2009).

2 Keller, H., Goy, R. & Kane, S. Validity and reliability of SCREEN II (Seniors in the community: risk evaluation for eating and nutrition, Version II). *European Journal of Clinical Nutrition* **59**, 1149-1157 (2005).

3 Herdman, M. *et al.* Development and preliminary testing of the new five-level version of EQ-5D (EQ-5D-5L). *Quality of life research* **20**, 1727-1736 (2011).

4 Rabin, R. & Charro, F. d. EQ-SD: a measure of health status from the EuroQol Group. *Annals of medicine* **33**, 337-343 (2001).
