# Supplementary material for: Derivation and validation of an epigenetic frailty risk score in population-based cohorts of older adults
Source: Nat Commun. 2022 Sep 7;13:5269. doi: 10.1038/s41467-022-32893-x (PMC9450828; doi:10.1038/s41467-022-32893-x)
Supplement: Supplementary file 3 — Description of Additional Supplementary Files [file 41467_2022_32893_MOESM3_ESM.pdf]

Description of Additional Supplementary Files

File Name: Supplementary Data 1
Description: Associations of the 65 identified CpGs with frailty in the validation panel (subset II)

File Name: Supplementary Data 2
Description: Gene functions and related diseases of the identified loci.

File Name: Supplementary Data 3
Description: Results of mQTL analysis on CpGs included in eFRS.

File Name: Supplementary Data 4
Description: Proportions of status of deficits included in the frailty index calculation in ESTHER at each follow-up.
