# Supplementary material for: Derivation and validation of an epigenetic frailty risk score in population-based cohorts of older adults
Source: Nat Commun. 2022 Sep 7;13:5269. doi: 10.1038/s41467-022-32893-x (PMC9450828; doi:10.1038/s41467-022-32893-x)
Supplement: Supplementary file 8 — Reporting Summary [file 41467_2022_32893_MOESM8_ESM.pdf]

nature portfolio

Corresponding author(s): Hermann Brenner

Last updated by author(s): Aug 10, 2022

Reporting Summary

Nature Portfolio wishes to improve the reproducibility of the work that we publish. This form provides structure for consistency and transparency in reporting. For further information on Nature Portfolio policies, see our Editorial Policies and the Editorial Policy Checklist.

Statistics

For all statistical analyses, confirm that the following items are present in the figure legend, table legend, main text, or Methods section.

n/a	Confirmed	
☐	☒	The exact sample size (*n*) for each experimental group/condition, given as a discrete number and unit of measurement
☐	☒	A statement on whether measurements were taken from distinct samples or whether the same sample was measured repeatedly
☐	☒	The statistical test(s) used AND whether they are one- or two-sided<br>*Only common tests should be described solely by name; describe more complex techniques in the Methods section.*
☐	☒	A description of all covariates tested
☐	☒	A description of any assumptions or corrections, such as tests of normality and adjustment for multiple comparisons
☐	☒	A full description of the statistical parameters including central tendency (e.g. means) or other basic estimates (e.g. regression coefficient) AND variation (e.g. standard deviation) or associated estimates of uncertainty (e.g. confidence intervals)
☐	☒	For null hypothesis testing, the test statistic (e.g. *F*, *t*, *r*) with confidence intervals, effect sizes, degrees of freedom and *P* value noted<br>*Give P values as exact values whenever suitable.*
☒	☐	For Bayesian analysis, information on the choice of priors and Markov chain Monte Carlo settings
☒	☐	For hierarchical and complex designs, identification of the appropriate level for tests and full reporting of outcomes
☐	☒	Estimates of effect sizes (e.g. Cohen's *d*, Pearson's *r*), indicating how they were calculated

Our web collection on statistics for biologists contains articles on many of the points above.

Software and code

Policy information about availability of computer code

Data collection	No software used in data collection
Data analysis	R programming (version 4.0.1) package 'glmnet (version 4.1-4)': https://cran.r-project.org/web/packages/glmnet/index.html<br>Annotations of genes related to CpGs: http://emea.support.illumina.com/array/array_kits/infinium-methylationepic-beadchip-kit/downloads.html#<br>Metascape online tool: https://metascape.org<br>mQTLdb: http://www.mqtldb.org<br>SAS 9.4 (SAS Institute, Cary, NC)<br>SAS codes for statistical analysis are available upon reasonable request (X.Li, li.xiangwei@foxmail.com).

For manuscripts utilizing custom algorithms or software that are central to the research but not yet described in published literature, software must be made available to editors and reviewers. We strongly encourage code deposition in a community repository (e.g. GitHub). See the Nature Portfolio guidelines for submitting code & software for further information.

Data

Policy information about availability of data

All manuscripts must include a data availability statement. This statement should provide the following information, where applicable:

- Accession codes, unique identifiers, or web links for publicly available datasets
- A description of any restrictions on data availability
- For clinical datasets or third party data, please ensure that the statement adheres to our policy

All relevant data supporting the key findings of this study are available within the article and its supplementary information files. Due to ethical and legal restrictions, individual-level data of the two cohorts (ESTHER and KORA-age) cannot be made publicly available. Data are available upon request to X.Li (li.xiangwei@foxmail.com) and subject to local rules and regulations. This includes submitting a proposal to the management team, where upon approval, analysis needs to be done on a local server with protected access, complying with General Data Protection Regulation. Requests will be responded in 60 working days. Annotations of genes of frailty related CpGs were downloaded from (http://emea.support.illumina.com/array/array_kits/infinium-methylationepic-beadchip-kit/downloads.html#). The underlying roles of these genes are available at (https://metascape.org). Results of mQTL were downloaded from the online tool mQTLdb (http://www.mqtldb.org). The original files generated from the three websites have been deposited at (https://figshare.com/articles/dataset/mQTLdb_Metascape_Annotations/20468985).

Human research participants

Policy information about studies involving human research participants and Sex and Gender in Research.

Reporting on sex and gender	The findings in our study are not apply to only one sex. Sex was not considered in the study design.<br>No gender-based analyses were conducted. All participants were randomly selected from population-based cohort study.
Population characteristics	In ESTHER, mean age was approximately 62 years, and a slight majority of participants were women. In KORA-age, the mean age was about 76 years and half of the participants were females.
Recruitment	The samples were randomly selected from two prospective, population-based cohort studies. They were observed comparable major sociodemographic characteristics to the total population living in the Germany.
Ethics oversight	The ESTHER study was approved by the ethics committees of the medical faculty of Heidelberg University and of the medical board of the state of Saarland. The KORA-Age study was approved by the Ethics Committee of the Bavarian Medical Association (EK No 08064). All ESTHER and KORA-Age participants provided written informed consent.

Note that full information on the approval of the study protocol must also be provided in the manuscript.

Field-specific reporting

Please select the one below that is the best fit for your research. If you are not sure, read the appropriate sections before making your selection.

☒ Life sciences ☐ Behavioural & social sciences ☐ Ecological, evolutionary & environmental sciences

For a reference copy of the document with all sections, see nature.com/documents/nr-reporting-summary-flat.pdf

Life sciences study design

All studies must disclose on these points even when the disclosure is negative.

Sample size	ESTHER study is an ongoing prospective, population-based cohort study of 9940 older adults conducted in the federal state of Saarland, Germany. KORA-Age study is a population-based cohort study conducted in the region of Augsburg, Southern Germany.<br>4 subsets with 3276 study subjects were randomly selected from the two cohorts for DNAm assessment.<br>No sample size calculation was performed. The sample size was made as large as it could be based on the availability of suitable material for DNAm assessment.
Data exclusions	No data was excluded.
Replication	The findings were confirmed in another independent population-based cohort study, the KORA-Age study
Randomization	It is a observational study and randomization was not applicable
Blinding	It is a observational study and no need to blind the participants.

Reporting for specific materials, systems and methods

We require information from authors about some types of materials, experimental systems and methods used in many studies. Here, indicate whether each material, system or method listed is relevant to your study. If you are not sure if a list item applies to your research, read the appropriate section before selecting a response.

Materials & experimental systems

n/a	Involved in the study
☒	☐ Antibodies
☒	☐ Eukaryotic cell lines
☒	☐ Palaeontology and archaeology
☒	☐ Animals and other organisms
☒	☐ Clinical data
☒	☐ Dual use research of concern

Methods

n/a	Involved in the study
☒	☐ ChIP-seq
☒	☐ Flow cytometry
☒	☐ MRI-based neuroimaging
